# Effect of the ionic product of bioglass 60s on osteoblastic activity in canines

**DOI:** 10.1186/s12917-015-0558-7

**Published:** 2015-09-30

**Authors:** Endrigo G L Alves, Rogéria Serakides, Isabel R. Rosado, Marivalda M. Pereira, Natália M. Ocarino, Humberto P. Oliveira, Alfredo M. Góes, Cleuza M F Rezende

**Affiliations:** Veterinary Medicine Program at the University of Uberaba (Universidade de Uberaba - UNIUBE), Uberaba, Brazil; Center for Stem Cells and Animal Cell Therapy (Núcleo de Células Tronco e Terapia Celular Animal - NCT-TCA), Department of Medicine and Surgery, Veterinary School of the Federal University of Minas Gerais (Universidade Federal de Minas Gerais - UFMG), Belo Horizonte, Brazil; Laboratory of Biomaterials of the Department of Metallurgic and Materials Engineering at UFMG, Belo Horizonte, Brazil; Department of Biochemistry and Immunology of the Institute of Biological Sciences at UFMG, Belo Horizonte, Brazil

**Keywords:** Orthopaedics, Biomaterials, Bone substitute, Osteoblasts

## Abstract

**Background:**

The objective of the present study was to evaluate the effect of the ionic product (***IP***) of BG60S on osteoblastic activity. The following media groups were created: ***DMEM***, which is formed by osteoblasts in basal medium; ***IP DMEM***, which is formed by osteoblasts in *IP* with basal medium; ***OST***, which is formed by osteoblasts in osteogenic medium; and ***IP OST***, which is formed by osteoblasts in *IP* with osteogenic medium. The osteoblasts were cultivated in an incubator at 37 °C and 5 % CO_2_ for 7, 14 and 21 days. After each period, the alkaline phosphatase (AP) activity, mineralised area per field and expression of osterix (OSX), bone sialoprotein (BSP), osteonectin (ON) and osteocalcin (OC) were evaluated by reverse transcription (RT)-PCR.

**Results:**

The *IP* significantly increased the AP activity in the *IP DMEM* group at 7 and 14 days and reduced the AP activity in the *IP OST* group at 14 and 21 days relative to their respective controls (*DMEM* and *OST*). The groups that received the *IP* displayed a significant increase in the percentage of mineralised area per field and more advance maturation of the extracellular matrix relative to those that did not receive *IP*. The *IP* significantly increased the expression of OSX, BSP and ON in osteoblast cultures maintained in *IP DMEM* compared with the control (*DMEM*) for the majority of studied periods. In osteogenic medium, *IP* also significantly increased OSX, BSP, ON and OC expression compared with the control (*OST*) for the majority of studied periods.

**Conclusions:**

The *IP* of BG60S alters the gene expression of canine osteoblasts, favouring the synthesis and mineralisation of the extracellular matrix.

## Background

Bioglass 60S (BG60S) has a molar composition of 4 % P_2_O_5_, 36 % CaO and 60 % SiO_2_, is a biomaterial with favourable characteristics for bone regeneration [1] and displays beneficial effects on the repair of bone defects in dog jaws [2]. But nothing is known about the effect of this biomaterial on the activity of dog osteoblasts, especially as regards the production of extracellular matrix and osteogenic differentiation. Generally speaking, when the bioglass is implanted on the bone tissue, it provides a framework for migration and colonization by osteoblasts, and apart from this, it reacts with the interstitial fluids, releasing ions, mainly calcium and silicon [3], which are, probably, primarily responsible for the biological effect.. These ions may favour osteogenic proliferation [4, 5] and differentiation *in vitro* [5, 6]. However, the ideal chemical composition of bioglass that releases the ideal combination and concentration of ions to maximise the bone regeneration process upon implantation is not well understood. In an *in vitro* study with rat osteoblasts, BG60S exhibited satisfactory Ca and Si release rates, which favours cellular proliferation [3]. However, the effects of this biomaterial on canine osteoblast activity are unknown. Osteoblasts perform a crucial function in the bone regeneration process by producing and secreting the extracellular bone matrix. The objective of the present study was to investigate the hypothesis that the ionic product (*IP*) of BG60S will increase the synthesis and mineralisation of the extracellular matrix by evaluating the effect of the *IP* on canine osteoblastic activity.

## Results

### Concentrations of Si and Ca ions in cell culture media

When added to cell culture media, BG60S was partially dissolved, causing a significant increase in the concentration of Si and Ca ions. The concentration of Si ions was 40.91 mg/L in *IP DMEM* medium (which is formed by osteoblasts in *IP* with basal medium), 41.28 mg/L in *IP OST* medium (which is formed by osteoblasts in *IP* with osteogenic medium) and lower than 1 mg/L in the media with no contact with BG60S (*DMEM* and *OST,* which are formed by osteoblasts in basal medium and osteoblasts in osteogenic medium, respectively). The concentration of Ca ions was 96.5 mg/L in *DMEM* medium, 87.5 mg/L in *OST* medium, 169 mg/L in *IP DMEM* medium and 156.5 mg/L in *IP OST* medium. Hence, BG60S increased the concentration of Ca ions by 75 and 79 % in basal and osteogenic media, respectively.

### Alkaline phosphatase activity

**Fig. 1:**
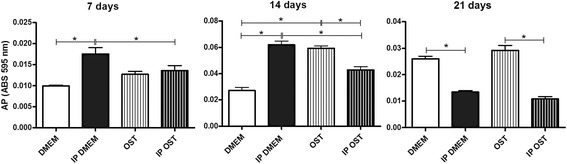
Mean and standard deviation of the alkaline phosphatase activity in canine osteoblast cultures maintained in basal medium (*DMEM*), ionic product in basal medium (*IP DMEM*), osteogenic medium (*OST*) and ionic product in osteogenic medium (*IP OST*) at 7, 14 and 21 days. *(*P* < 0.05)

The osteoblast cultures maintained in *IP DMEM* exhibited higher alkaline phosphatase (AP) activity than those maintained in *DMEM* at 7 and 14 days and lower AP at 21 days (Fig. 1). The *IP OST* group displayed lower AP activity than group *OST* at 14 and 21 days, and no difference was observed at 7 days (Fig. 1). Higher AP activity was observed in the *OST* group relative to the *DMEM* group at all times studied. The cultures maintained in *IP DMEM* displayed higher activity than those in *IP OST* at 7 and 14 days, and no difference was observed at 21 days (Fig. 1).

### Evaluation of the mineralised matrix synthesis

**Fig. 2:**
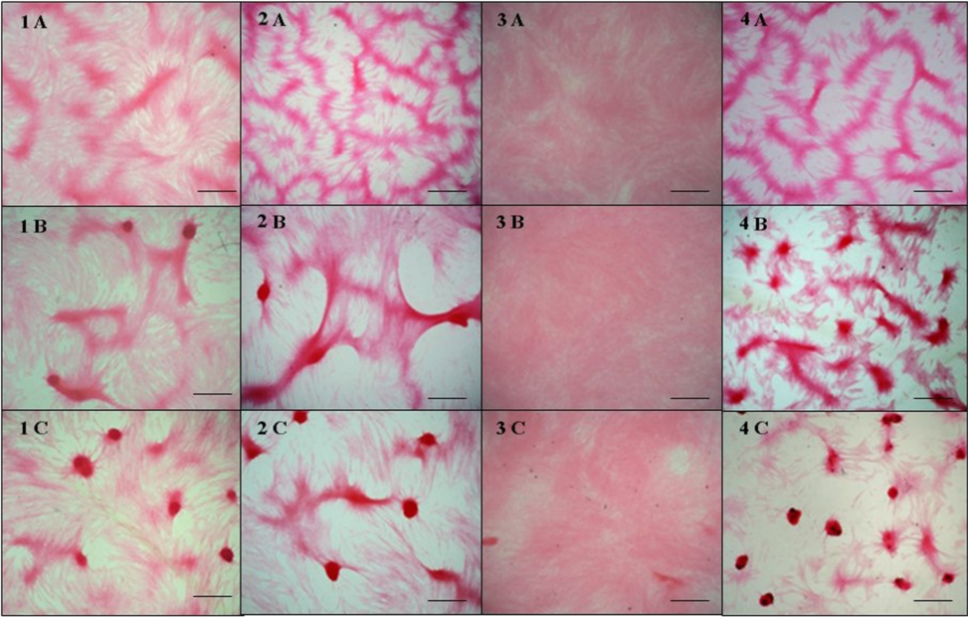
Canine osteoblast culture maintained in basal medium (*DMEM*) (1), ionic product in basal medium (*IP DMEM*) (2), osteogenic medium (*OST*) (3) and ionic product in osteogenic medium (*IP OST*) (4) for 7 (**a**), 14 (**b**), and 21 (**c**) days. The mineralisation nodules dyed by the Von Kossa technique can be observed. The groups with *IP*, *IP DMEM* (2) and *IP OST* (4), exhibited larger mineralised area per field and greater maturation of the extracellular matrix than their respective controls, *DMEM* (1) and *OST* (3). Bar = 350 μm

**Fig. 3:**
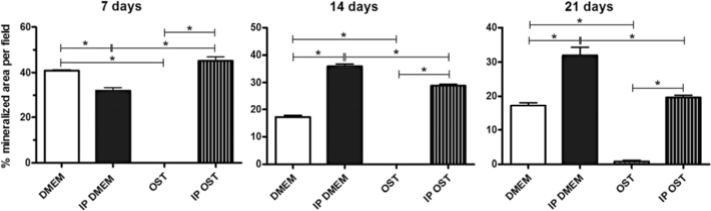
Mean percentage and standard deviation of the mineralised area per field in canine osteoblast cultures cultivated in basal medium (*DMEM*), ionic product in basal medium (*IP DMEM*), osteogenic medium (*OST*) and ionic product in osteogenic medium (*IP OST*) for 7, 14 and 21 days. *(*P* < 0.05)

The osteoblast cultures maintained in *IP DMEM* displayed a higher mineralised area per field than that of cultures maintained in *DMEM* at 14 and 21 days and lower mineralised area per field at 7 days (Figs. 2 and 3). The *IP OST* group displayed a higher mineralised area by field than the *OST* group at 7, 14 and 21 days of culture (Figs. 2 and 3). The *IP DMEM* and *IP OST* groups exhibited a higher mineralisation of the matrix than did the *DMEM* and *OST* groups, respectively (Fig. 3). The *DMEM* group displayed a higher mineralised area by field than the *OST* group for all times studied (Figs. 2 and 3). The cultures maintained in *IP DMEM* displayed a larger mineralised area per field than that of cultures maintained in *IP OST* at 14 and 21 days and a smaller mineralised area per field at 7 days; however, the *IP OST* group exhibited a higher mineralisation of the extracellular matrix than did the *IP DMEM* group (Figs. 2 and 3).

### Quantification of gene transcriptions for OSX, BSP, ON and OC

**Fig. 4:**
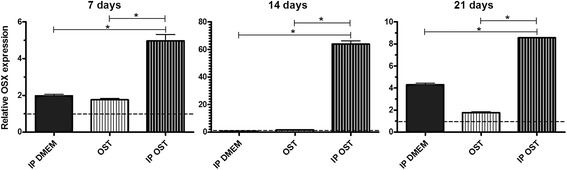
Mean and standard deviation of gene transcription for osterix (OSX) by RT-PCR in canine osteoblast cultures maintained in basal medium (*DMEM*), ionic product in basal medium (*IP DMEM*), osteogenic medium (*OST*) and ionic product in osteogenic medium (*IP OST*) for 7, 14 and 21 days. The dotted line represents the expression of canine osteoblasts in basal medium (*DMEM*). *(*P* < 0.05)

The osteoblast cultures maintained in *IP DMEM* exhibited higher osterix (OSX) expression than those maintained in *DMEM* at 7 and 21 days, and no difference was observed at 14 days (Fig. 4). Group *IP OST* displayed greater OSX expression than group *OST,* at 7, 14, and 21 days of culture (Fig. 4). At 7 and 21 days of culture, higher OSX expression was observed in group *OST* relative to group *DMEM*, and at 14 days, no difference was observed (Fig. 4). The cultures maintained in *IP OST* exhibited greater OSX expression than those maintained in *IP DMEM* at 7, 14, and 21 days of culture (Fig. 4).

**Fig. 5:**
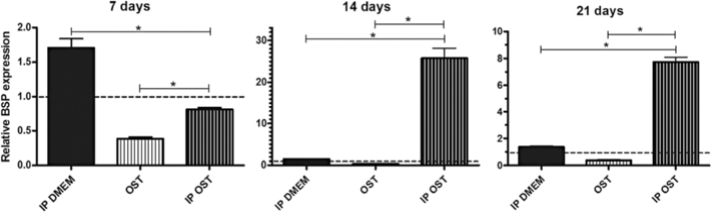
Mean and standard deviation of gene transcription for bone sialoprotein (BSP) by RT-PCR in canine osteoblast cultures maintained in basal medium (*DMEM*), ionic product in basal medium (*IP DMEM*), osteogenic medium (*OST*) and ionic product in osteogenic medium (*IP OST*) for 7, 14 and 21 days. The dotted line represents the expression of canine osteoblasts maintained in basal medium (*DMEM*). *(*P* < 0.05)

The osteoblast cultures maintained in *IP DMEM* displayed greater bone sialoprotein (BSP) expression than those maintained in *DMEM* at 7 and 21 days, and no difference was observed at 14 days (Fig. 5). The *IP OST* group displayed higher BSP expression than did the *OST* group at 7, 14, and 21 days of culture (Fig. 5). Lower BSP expression was observed in the *OST* group compared with the *DMEM* group at 7, 14 and 21 days (Fig. 5). The cultures maintained in *IP OST* exhibited greater BSP expression than those maintained in *IP DMEM* at 14 and 21 days of culture and lower BSP expression at 7 days (Fig. 5).

**Fig. 6:**
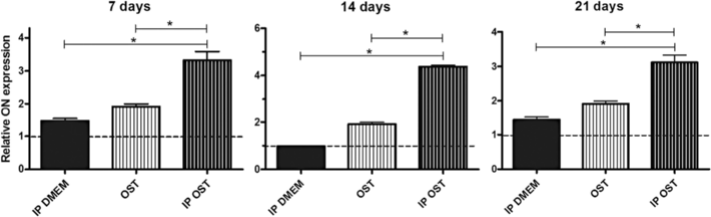
Mean and standard deviation of gene transcription for osteonectin (ON) by RT-PCR in canine osteoblast cultures maintained in basal medium (*DMEM*), ionic product in basal medium (*IP DMEM*), osteogenic medium (*OST*) and ionic product in osteogenic medium (*IP OST*) for 7, 14 and 21 days. The dotted line represents the expression of canine osteoblasts in basal medium (*DMEM*). *(*P* < 0.05)

The osteoblast cultures maintained in *IP DMEM* displayed higher osteonectin (ON) expression than those maintained in *DMEM* at 7 and 21 days, and no difference was observed at 14 days (Fig. 6). The *IP OST* group exhibited greater ON expression than did the *OST* group at 7, 14, and 21 days of culture (Fig. 6). Higher ON expression was found in the *OST* group relative to the *DMEM* group at 7, 14, and 21 days (Fig. 6). The cultures maintained in *IP OST* exhibited greater ON expression than those maintained in *IP DMEM* at 7, 14, and 21 days of culture (Fig. 6).

**Fig. 7:**
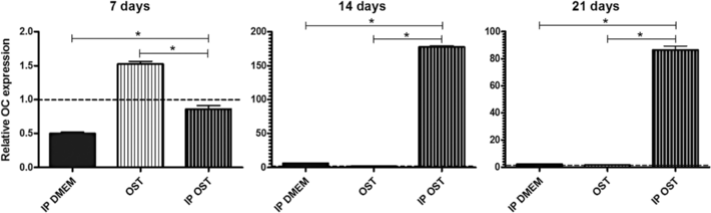
Mean and standard deviation of gene transcription for osteocalcin (OC) by RT-PCR in canine osteoblast cultures maintained in basal medium (*DMEM*), ionic product in basal medium (*IP DMEM*), osteogenic medium (*OST*) and ionic product in osteogenic medium (*IP OST*) for 7, 14 and 21 days. The dotted line represents the expression of canine osteoblasts maintained in basal medium (*DMEM*). *(*P* < 0.05)

The osteoblast cultures maintained in *IP DMEM* displayed lower osteocalcin (OC) expression than those maintained in *DMEM* at 7 days, and no difference was observed at 14 and 21 days (Fig. 7). The *IP OST* group displayed greater OC expression than the *OST* group at 14 and 21 days. At 7 days, the opposite was observed (Fig. 7), with greater OC expression observed in the *OST* group relative to the *DMEM* group, and no difference was observed at 14 and 21 days (Fig. 7). The cultures maintained in *IP OST* exhibited greater OC expression than those maintained in *IP DMEM* at 7, 14, and 21 days of culture (Fig. 7).

## Discussion

The biological effect of biomaterials can be evaluated by their dissolution product [3] because the ions liberated during dissolution contribute for the biological effect [7]. The increased concentration of Si (more than 400 %) and Ca ions (more than 75 %) from the dissolution of BG60S was expected; however, it was significantly higher than the values reported in the literature with bioglass of different compositions [8, 9]. This result indicates that the same type of biomaterial can produce different results according to the specific composition, which explains the various responses found in the literature.

The increase in AP activity in group *IP DMEM* relative to group *DMEM* at 7 and 14 days suggests a stimulating effect of the *IP* on the secreting activity of the osteoblast. AP is an enzyme that occurs in the membrane of the osteoblast, and it directly participates in the synthesis and mineralisation of bone matrix [10]. This enzyme is commonly used as a marker of osteogenic differentiation [11] and a parameter for the evaluation of the bone activity, and high AP activity also indicates high osteoblastic activity [7].

The significant increase of the extracellular matrix area and greater mineralisation of the extracellular matrix observed in cultures that received the *IP* shows the stimulating effect that *IPs* of BG60S have on osteoblasts with regard to matrix synthesis and mineralisation. The synthesis and mineralisation of the extracellular matrix are considered the most reliable and representative parameters of *in vitro* osteoblastic activity [12]. The predominant increase of OSX, BSP and ON expression in the cultures that received the *IP* relative to those that did not suggests that the *IP* of BG60S stimulates the synthesis and mineralisation of the extracellular matrix by increasing the expression of these proteins. OSX is a specific transcription factor of osteogenic differentiation [13, 14] that acts on the transformation of pre-osteoblasts into mature osteoblasts [10, 15]. Furthermore, OSX acts on the synthesis and mineralisation of bone matrix via the intranuclear receptor of vitamin D and on the inhibition of osteoblastic proliferation via the inhibition of Wnt (*Wingless-type MMTV integration site family*) signalling [13, 14]. BSP and ON are non-collagen proteins in bone matrix used as markers of osteogenic differentiation and osteoblastic activity [11, 16, 17]. These proteins are intimately related to the synthesis and mineralisation process of extracellular bone matrix [10]. Although the *IP* did not clearly increase OC expression, the increase in its expression in the *IP OST* group at 14 and 21 days suggests that the ions released by BG60S can also positively influence its expression. OC is a specific bone tissue glycoprotein expressed by osteoblasts and is considered the main non-collagen protein of bone matrix [16, 17]. The evaluation of its expression can be used for monitoring bone metabolism [15].

The beneficial effects of the *IP* of BG60S on osteoblastic activity are most likely caused by the Si and Ca ions released during its dissolution process. Studies with rat osteoblast cultures have demonstrated that Si can increase cellular activity and proliferation [18] as well as the expression of runx2, collagen type 1 and ON [19]. Furthermore, Si can favour the mineralisation of extracellular matrix [18]. Ca is another ion released during BG60S dissolution, and it can increase the activity and proliferation of human mesenchymal stem cells [4, 5]. Studies have indicated that extracellular Ca can increase osteopontin, OC, BSP and bone morphogenetic protein-2 (BMP 2) expression in human mesenchymal stem cells [4]. Additionally, Ca favours the synthesis and mineralisation of extracellular matrix by human mesenchymal stem cells [5]. The previously mentioned studies have indicated that Ca favours *in vitro* osteogenesis; however, little is known of the mechanisms involved. The increase in extracellular Ca can increase the expression of osteopontin and BSP in mouse pre-osteoblasts, an effect mediated by L-type Ca channels and the Ca/calmodulin-dependent protein kinase 2 (CaM-CaMK2) signalling pathway [6].

With the limitations of the current study, there is only assessment of the 60S bio-glass in the form of its ionic product. Although assessment of the product of dissolving the 60S bio-glass may bring important information concerning its form of action, the biological response of a biomaterial may also be influenced by the size, form, microstructure, texture and topography of the implant surface. Thus, it is important to carry out complementary studies that may assess all these variables, allowing for a better understanding as to how each one of them influences its biological response.

## Conclusion

The *IP* of BG60S alters the gene expression of canine osteoblasts, favouring the synthesis and mineralisation of extracellular matrix.

## Methods

This study was performed according to the international norms for animal welfare and approved by the UFMG Animal Experimentation Ethics Committee (protocol n. 157/2009).

### Biomaterial and ionic product

BG60S with a molar composition 4 % P_2_O_5_ 36 % CaO 60 % SiO_2_ was produced with 133.8 mL tetraethyl orthosilicate (TEOS), 13.6 mL triethyl phosphate (TEP), 97.9 mL deionised water, 16.3 mL 2 N nitric acid solution and 85.01 g Ca nitrate [Ca(NO_3_)_2_ 4H_2_O] in accordance with the protocol described by Coelho et al. [1]. The biomaterial was incubated at 60 °C for 72 h for gelation and maturation and then remained at 60 °C for 72 h for drying, which was followed by gradual temperature increase of 10 °C every 24 h until reaching 120 °C. After drying, the sample was milled, and the grain size fraction smaller than 137 μm was separated. The elimination of possible residues was performed by heating in a muffle furnace at 700 °C for 6 h and applying a heating and cooling rate of 1 °C per minute. To obtain the *IP*, 6 g biomaterial was added to 1 L low-glucose Dulbecco’s modified Eagle’s medium (Gibco, CA, USA) containing gentamicin (60 μg/L), penicillin (100 U/mL), streptomycin (100 μg/mL) and amphotericin (25 μg/mL) (PSA, Sigma-Aldrich, USA). The mixture was homogenised and incubated for 12 h at 6 °C. After incubation, the suspension was filtered using a 22 μm membrane, and the pH was adjusted to 7.2, which resulted in the *IP* of BG60S.

### Concentrations of Si and Ca ions in the cell culture media

Prior to the use of the culture media with canine osteoblasts, the concentration of Si and Ca ions in the media was measured. The atomic absorption spectrometry technique (GBC-Avanta) was used according to the standard addition method to determine the ion concentration.

### Preparation and cultivation of osteoblasts and formation of the experimental groups

A canine osteoblast culture (CnOb - canine osteoblasts, Cell Application, CA, USA) was used to evaluate the effect of the *IP* of BG60S. The culture was defrosted, and then the pellet was plated and cultivated in T75 flaks with low-glucose *DMEM* (Gibco, CA, USA) containing gentamicin (60 μg/L), penicillin (100 U/mL), streptomycin (100 μg/mL) and amphotericin (25 μg/mL) (PSA, Sigma-Aldrich, USA) and enriched with 10 % foetal bovine serum (FBS - Soralis, Brazil) [basal medium]. The flasks were maintained in an incubator at 37 °C and 5 % CO_2_. The culture medium was changed twice per week, and when 80–90 % confluence was achieved, the cells were subcultured. On the third subculture after obtaining 80–90 % confluence, the osteoblasts were used for the evaluation of the *IP*, and the following groups were formed: ***DMEM***, ***IP DMEM*** [*IP* enriched with 10 % FBS], ***OST*** [basal medium enriched with ascorbic acid (50 μg/mL), ß-glycerophosphate (10 mM) (Sigma-Aldrich, USA), dexamethasone (0.1 μM) (Aché, Brazil) and 10 % FBS] and ***IP OST*** [*IP* enriched with ascorbic acid (50 μg/mL), ß-glycerophosphate (10 mM), dexamethasone (0.1 μM) and 10 % FBS].

The osteoblasts were cultivated in quadruplicate at 37 °C and 5 % CO_2_ for 7, 14 and 21 days with *DMEM, IP DMEM, OST* or *IP OST*. Subsequently, the AP activity, mineralised area per field and the relative expression of OSX, BSP, ON and OC were evaluated in each group by reverse transcriptase (RT)-PCR.

### Alkaline phosphatase activity

For the evaluation of AP activity, the osteoblasts of each group were cultivated separately in 24-well plates. At the end of each period, the cultures were washed with phosphate buffered saline (PBS - 0.15 molar). To each well, 200 μL of 5-Bromo-4-Chloro-3-Indolyl phosphate/nitroblue tetrazolium salt (BCIT/NBT) solution (Zymed Laboratories, USA) was added. The samples were incubated for 2 h in an oven at 37 °C and 5 % CO_2_ and observed under an optical microscope before the addition of 200 μL of sodium dodecyl sulphate (SDS)-10 % HCl. These samples were placed in an oven overnight at 37 °C and 5 % CO_2_, and then 100 μL from each well was transferred to 96-well plates and read by a spectrophotometer at 595 nm [11].

### Evaluation of mineralised matrix synthesis

To evaluate the percentage of mineralised matrix per field, the osteoblasts of each group were cultivated separately in 6-well plates with cover slips (Sarstedt, USA). After each evaluation period, the osteoblasts were fixed with 70 % ethanol for 24 h and dyed using the Von Kossa method. The percentage of mineralised matrix per field was determined with an ocular micrometer containing a 121-point grid, with 25 fields and a 4× objective [11].

### Quantification of gene transcription for OSX, BSP, ON and OC

For the relative quantification of gene transcription for OSX, BSP, ON and OC by RT-PCR, the osteoblasts of each group were cultivated in quadruplicate in T25 flasks. At the end of each evaluation period, the total RNA was extracted from the cultures of each bottle with Trizol (Invitrogen, USA) following the manufacturer’s protocol. The RNA was solubilised in RNase-free diethylpyrocarbonate (DEPC) water (Invitrogen, USA) and immediately stored at −80 °C. The RNA concentration was determined by an absorbance reading at 260/280 nm by spectrophotometry. Reverse transcription reactions were performed using Kit SuperScript™ III Platinum® Two-Step (Invitrogen, USA). Total RNA (1 μg) was used for cDNA synthesis, with a final volume of 20 μL. The PCR reactions were performed in real-time using 2 μg of cDNA, 5 pM of each primer and 12.5 μL of the reagent SYBR Green at a final volume of 25 μL reaction per well, in a 7500 Real-Time PCR System (Applied Biosystems, USA). The parameters used for amplification were 50 °C for 120 s, 95 °C for 150 s and 45 cycles, 95 °C for 15 s and 60 °C for 30 s. The primers were researched in the literature or designed based on the sequence of *Canis familiaris* mRNA (Table 1). The gene expression was calculated with the method 2^-∆∆CT^, and the results obtained for each group were quantitatively compared after the normalisation based on the expression of *Canis familiaris* glyceraldehyde 3-phosphate dehydrogenase (GAPDH)*.* The expression levels obtained in osteoblastic cultures cultivated in basal medium were used as expression standards to calculate the relative expression of each transcription.

**Table 1:** Genes and nucleotide sequence of primers used for RT-PCR

Gene	Primer	Annealing temperature (°C)	Product size
Reference or Accession No.	(5′ to 3′ nucleotide sequences)		(base pairs)
OSX	F- ACGACACTGGGCAAAGCAG	60	285
Neupane et al. [16]	R- CATGTCCAGGGAGGTGTAGAC		
BSP	F- TTGCTCAGCATTTTGGGAAT	60	295
Vieira et al. [17]	R- AACGTGGCCGATACTTAAAGAC		
ON	F- GCCTTGGCAGCCCCTCAACA	60	108
(XM_849889.1)	R- CACCTGCACGGGGTTGGCTC		
OC	F- GAGGGCAGCGAGGTGGTGAG	62	134
Neupane et al. [16]	R- TCAGCCAGCTCGTCACAGTTGG		
GAPDH	F- CCATCTTCCAGGAGCGAGGAT	60	97
Vieira et al. [17]	R- TTCTCCATGGTGGTGAAGAC		

#### Statistical analysis

An analysis of variance (ANOVA) was performed, and the mean and standard deviation were determined for each variable. The means were compared by Student’s *t*-test using the program Graphpad Instat 3 (GraphPad Software Inc., USA). Differences were considered significant at *P* < 0.05.

## Abbreviations

AP: Alkaline phosphatase
ANOVA: Analysis of variance
BCIT: 5-Bromo-4-Chloro-3-Indolyl phosphate
BMP-2: Bone morphogenetic protein-2
BSP: Bone sialoprotein
Ca: Calcium
cDNA: Complementary DNA
CETEA: Animal experimentation ethics committee (Comitê de Ética em Experimentação Animal)
DEPC: Diethylpyrocarbonate
DMEM: Dulbecco’s modified Eagle’s medium (basal medium)
FBS: Foetal bovine serum
GAPDH: Glyceraldehyde-3-phosphate dehydrogenase
mRNA: RNA messenger
NBT: Nitroblue tetrazolium salt
NCT-TCA: Centre for Stem Cells and Animal Cell Therapy (Núcleo de Células Tronco e Terapia Celular Animal)
OC: Osteocalcin
OSX: Osterix
IP: Ionic product
IP DMEM: Ionic product in basal medium
IP OST: Ionic product in osteogenic medium
PSA: Penicillin, streptomycin and amphotericin
qRT-PCR: Quantitative reverse transcription polymerase chain reaction
RNA: Ribonucleic acid
RNase: Ribonuclease enzyme
RUNX-2: Runt-related transcription factor 2
SDS: Sodium dodecyl sulphate
Si: Silicon
TEOS: Tetraethylorthosilicate
TEP: Triethylphosphate
UFMG: Federal University of Minas Gerais (Universidade Federal de Minas Gerais)
UNIUBE: University of Uberaba (Universidade de Uberaba)
Wnt: *Wingless-type MMTV integration site family*

## References

[1] Coelho MB, Pereira MM (2005). Sol–gel synthesis of bioactive glass scaffolds for tissue engineering: effect of surfactant type and concentration. J. Biomed. Mater. Res. B Appl. Biomater..

[2] Dutra CE, Pereira MM, Serakides R, Rezende CMF (2008). In vivo evaluation of bioactive glass foams associated with platelet-rich plasma in bone defects. J Tissue Eng Regen Me.

[3] Valério P, Pereira MM, Goes AM, Leite MF (2004). The effect of ionic products from bioactive glass dissolution on osteoblast proliferation and collagen production. Biomaterials.

[4] Barradas AMC, Fernandes HAM, Groen N, Chai YC, Schrooten J, van de Peppel J, van Leeuwen JP, van Bitterswijk CA, de Boer J (2012). A calcium-induced signalling cascade leading to osteogenic differentiation of human bone marrow-derived mesenchymal stromal cells. Biomaterials.

[5] Mccullen SD, Zhan J, Onorato ML, Bemacki SH, Loboa EG (2010). Effect of varied ionic calcium on human adipose-derived stem cell mineralization. Tissue Eng. Part A.

[6] Jung GY, Park YJ (2010). Effects of HA-released calcium ions on osteoblast differentiation. J Mater Sci Mater Med.

[7] Li HW, Sun JY (2011). Effects of dicalcium silicate coating ionic dissolution products on human mesenchymal stem-cell proliferation and osteogenic differentiation. J Int Med Res.

[8] Tsigkou O, Jones JR, Polak JM, Stevens MM (2009). Differentiation of fetal osteoblasts and formation of mineralized bone nodules by 45S5 Bioglass conditioned medium in the absence of osteogenic supplements. Biomaterials.

[9] Alno N, Jegoux F, Pellen-Mussi P, Tricot-Doleux S, Oudadesse H, Cathelineau G, de Mello G (2010). Development of a three-dimensional model for rapid evaluation of bone substitutes in vitro: Effect of the 45S5 bioglass. J. Biomed. Mater. Res. A.

[10] Franceschi RT, Ge C, Xiao G, Roca H, Jiang D (2009). Transcriptional regulation of osteoblasts. Cells Tissues Organs.

[11] Alves EGL, Serakides R, Boeloni JN, Rosado IR, Ocarino NM, Oliveira HP, Góes AM, Rezende CMF (2014). Comparison of the osteogenic potential of mesenchymal stem cells from the bone marrow and adipose tissue of young dogs. BMC Vet Res 2014.

[12] Bruedigan C, Driel MV, Koedam M, Peppel JV, van der Eerden BC, Eijken M, van Leeuwen JP (2011). Basic techniques in human mesenchymal stem cell cultures: differentiation into osteogenic and adipogenic lineages, genetic perturbations, and phenotypic analyses. Curr Protoc Stem Cell Biol.

[13] Long F (2012). Building strong bones: molecular regulation of the osteoblast lineage. Nat Rev Mol Cell Biol.

[14] Zhang C (2012). Molecular mechanisms of osteoblast-specific transcription factor osterix effect on bone formation. Beijing Da Xue Xue Bao.

[15] Nishimura R, Hata K, Matsubara T, Wakabayashi M, Yoneda T (2012). Regulation of bone and cartilage development by the network between BMP signalling and transcription factors. J Biochem..

[16] Neupane M, Chang C, Kiupel M, Yuzbasiyan-Gurkan V (2008). Isolation and characterization of canine adipose-derived mesenquimal stem cells. Tissue Eng A.

[17] Vieira NM, Brandalise V, Zucconi E, Secco M, Strauss BE, Zatz M (2010). Isolation, characterization, and differentiation potential of canine adipose-derived stem cells. Cell Transplant.

[18] Shie MY, Ding SJ, Chang HC (2011). The role of silicon in osteoblast-like cell proliferation and apoptosis. Acta Biomater.

[19] Varanasi VG, Leong KK, Dominia LM, Jue SM, Loomer PM, Marshall GW (2012). Si and Ca individually and combinatorially target enhanced MC3T3-E1 subclone 4 early osteogenic marker expression. J. Oral Implantol..

